# “What happens when you get corona?”: Children’s questions and parental responses about the COVID-19 pandemic

**DOI:** 10.1371/journal.pone.0330506

**Published:** 2025-08-18

**Authors:** Graciela Trujillo Hernández, David Menendez, Seung Heon Yoo, Rebecca E. Klapper, Maria H. Schapfel, Kailee A. Sowers, Victoria E. Welch, Karl S. Rosengren

**Affiliations:** 1 Department of Psychology and Department of Brain and Cognitive Science, University of Rochester, Rochester, New York, United States of America; 2 Department of Psychology, University of California-Santa Cruz, Santa Cruz, California, United States of America; Carol Davila University of Medicine and Pharmacy, ROMANIA

## Abstract

Information discussed between parents and children provide a foundation for children's developing understanding of health and illness. Parents of 3-to-7-year-old children (N = 516, 62% female, 78% White) residing in the United States were recruited using Amazon’s Mechanical Turk during July 29^th^– August 10^th^, 2020. We asked parents to report three questions that their children had asked about the COVID-19 pandemic and asked them to report how they responded to those questions. Children’s questions focused on lifestyle changes (22%), while parental responses were often about the virus (23%). We examined the stability of content of children’s questions and parental responses between the first peak and second peak of infection and death rates due to COVID-19 in the United States. The topic of children’s questions and the types of parental responses shifted between the two peaks, such that parents during the second peak of the pandemic reported their children asking more frequently about the virus and preventive measures than children in the first peak. Meanwhile, parents during the second peak of infection and death rates were more focused on responding to their children’s questions with information about the virus. We used Latent Class Analysis to explore overall patterns in children’s questions and parents’ responses. For children’s questions, three latent classes were obtained: (1) the virus [39%], (2) the virus/lifestyle changes [21%], and (3) lifestyle changes/preventive measures [40%]. For parents’ responses three latent classes were found: (1) the virus/self-protection [54%], (2) reassurance/the virus [28%], and (3) simple yes/no answers without further explanation [17%]. These results suggest that children’s questions and parental responses can be captured in terms of a discrete number of latent classes.

## Introduction

At the start of the COVID-19 pandemic, the United States had one of the highest infection rates of COVID-19 across the globe [1]. COVID-19 cases caused considerable disturbance in the lives of families. Schools and daycare programs were compelled to halt operations. Parents were their children’s primary source of information about the pandemic, including information about the virus and safety measures put in place to protect them [2–5]. In this study, we examined whether there were patterns in children’s questions and parental responses about the COVID-19 pandemic. We focused on questions and responses reported at the second peak of infection and death rates in the United States (July 29^th^ – August 10^th^, 2020).

### Children’s understanding of illness

Research has shown that young children’s knowledge of biological phenomena is more sophisticated than previously assumed [6–9]. For example, Springer and Ruckel [10] have found that 4- to 5-year-old preschoolers reject the notion that illness is caused by misbehavior. In fact, they were more likely to believe that germs and poisons could cause illness. Other researchers [6–7] have also noted that, while preschoolers may not be able to explain how germs and viruses work, they do recognize that causes of illness are not always visible, obvious, or explainable. These findings suggest that young children cannot completely grasp the inner workings of illnesses.

The COVID-19 pandemic presented a unique opportunity to examine how children gain knowledge about complex and novel biological illnesses. One important source of children’s knowledge about illness is conversations with more knowledgeable others [11]. In this study we focus on how children’s questions and parental responses might foster children’s knowledge of the COVID-19 pandemic in the United States.

### Parent-child conversations

Social information plays a crucial role in the acquisition of knowledge within the domain of illness, as many concepts in the biology domain involve unobservable entities. Children must rely on more knowledgeable adults (e.g., parents) to inform their understanding of biological phenomena [12]. McIntosh and colleagues [13] have suggested that children learn about the causes of illnesses through their participation in everyday family activities. Parents, through everyday conversations and explanations they give to their children, shape their child’s knowledge. These conversations, in turn, may help shape the preventive behaviors children engage in. For this reason, parent-child conversations can play a pivotal role in encouraging children to think about health and illness.

The COVID-19 outbreak provided the opportunity for many parent-child conversations about health and illness to take place. As parents and children became more confined at home during the initial stages of the pandemic in the United States (approximately January 20^th^, 2020; [14]), parents became the primary source and, in some cases, the only source of information about the COVID-19 pandemic for their children.

### Children’s questions and parents’ responses

Children influence their own learning through the questions they ask their parents and other knowledgeable individuals. The responses these individuals provide to children in turn shape how children reason and act [15]. Chouinard [16] has argued that children’s questions are a driving force in children’s cognitive development. Ünlütabak and colleagues [17] suggested that children’s questions serve as a tool to help them learn from their conversation partners. As other researchers have noted [17–19], children do use questions to fill gaps in their knowledge.

In addition to the questions themselves, children’s learning is influenced by the content of parents’ responses. We argue that the types of answers children receive can shape their understanding of the biological realm. Parental responses to questions, then, promote learning if children’s questions are answered in an instructive manner. Although previous findings suggest that adults are quite responsive to children’s questions [16], parents do not always provide thorough responses. In some cases, parents may not be equipped with the necessary knowledge to provide children with detailed responses to their questions [20]. Other times, parents may find it unnecessary or inappropriate to answer children’s questions with intricate explanations [21]. Parents may also act as gatekeepers of information, managing, what, when, and how they provide information to their children [22]. As such, some parents may choose to limit the amount of information they share with the child based upon whether they think their child is old enough to know about the topic [23–25].

In the case of COVID-19, Menendez et al. [3] found that about half of parents shielded their children from information about the pandemic. When asked to explain why parents engaged in shielding, parents suggested that their child was too young and/or would not be able to cope with the information. Thus, parents may be using specific child characteristics (e.g., age, biology knowledge) to evaluate how much information is appropriate to be shared with their children.

Therefore, parent-child conversations are influenced by the child’s age. Children of school-age are more likely to possess knowledge that younger children have not yet acquired [19], and this may influence their questions and their parents’ responses. Research on chronic illnesses, such as cancer, identifies the age of a child as one of the main determinants of what type of information is conveyed by parents [23–25]. When faced with a complex and unknown disease like COVID-19 was at the time data was collected, parents may resort to using the child’s age and biology knowledge to determine what or how much information to share with the child.

### Children’s questions and parents’ responses about the COVID-19 pandemic

Research on the effects of the pandemic on families in the United States has been abundant. However, there have been few studies that specifically examined the information discussed between parents and children in relation to the COVID-19 pandemic. Nevertheless, the few studies that explicitly explored children’s questions and parental responses about the COVID-19 pandemic show that children use questions as a means of gathering information from their parents [3–4]. More specifically, past studies have found that around the first major peak of the pandemic children inquired more about lifestyle changes than COVID-19 itself [3–4]. Moreover, the type of questions children asked their parents about the events surrounding the pandemic has been shown to be influenced by some child factors, such as age and stress levels. For example, both Menendez et al. [3] and Haber et al., [4] found that younger, compared to older, children were more likely to ask questions regarding lifestyle changes. Similarly, parents who reported that their children were experiencing high levels of stress were more likely to inquire about the virus and the safety of their family and friends, and less likely to ask about lifestyle changes [3].

As for parental responses, Menendez et al. [3] did not find specific parental factors associated with the types of responses parents gave. Nevertheless, they found that many of the parents in their study provided responses to children’s questions about the pandemic that sought to reassure their children. They also found that some parents stressed the importance of having their child follow the appropriate measures to keep the child and the family safe. Others referred to authority figures (e.g., government officials, doctors, teachers) to answer their children’s questions about the COVID-19 pandemic. However, Menendez and colleagues [3] found that a substantial number of parental responses to children’s questions were yes/no/I don’t know answers with no further explanations. This suggests that parents may shield their children from potentially upsetting information.

Past research has focused on how children’s questions and parental responses looked like near the first major peak of COVID-19 infection and death rates (approximately around April 14^th^, 2020; [14]). The United States experienced multiple peaks of infection and death rates [26]. One reason for looking at the second peak was because the infection and death rates in the United States due to COVID-19 almost doubled from the first peak to the second (approximately around July-August 2020) [27–28]. This increase in infection and death rates may have increased the stress and perhaps the importance of adhering to preventive measures. It is also the case that during this time there was more information about the virus, including its ability to be transmitted by air and by asymptomatic individuals, than during its first peak [27]. This may have changed the content of children’s questions and how parents responded to those questions from peak one to peak two. Furthermore, the COVID-19 pandemic and our knowledge about the virus was constantly changing over time so, unlike other events that children ask questions about, the evolution of the pandemic may lead to changes in the content of children’s questions over time. Finally, during the period between the first and the second peak, the FDA expedited the use of rapid diagnostic testing and announced the availability of vaccines for older adults. [27]. Consequently, the second peak of the pandemic in the United States presented a unique opportunity to study how children’s questions and parents’ responses may have changed over time as we learned more about the virus and prevention strategies.

Previous studies looking at children’s questions and parents’ responses in general and more specifically about the COVID-19 pandemic have focused on individual children’s questions and parental responses to those questions [3–4]. Other studies focused on a limited set of questions and responses about the pandemic (e.g., what versus how questions; [29]). These studies show that parents respond to children’s questions in a variety of ways. Despite knowing that this range of responses exists, one question remains: do parents show a systematic pattern of responses? One goal of this study was to go beyond capturing a single snapshot of conversations between children and parents about the COVID-19 pandemic and examine whether there were changes in children’s questions and parental responses over time. The second goal was to go beyond individual questions and responses to explore whether there are any patterns in the questions and responses that reflect the overall concerns of the children and parents relating to the COVID-19 pandemic.

One way to examine this question is to use Latent Class Analysis (LCA) to determine whether children and parents can be classified as having characteristic patterns that reflect the main concerns they had related to the COVID-19 pandemic. LCA is statistical method used to classify individuals according to their pattern of behavior based on mutually exclusive and exhaustive groups called latent classes. This type of analysis has previously been used in developmental research, primarily in the area of developmental psychopathology [30–31]. To our knowledge, no one has used LCA as a method to examine specific patterns in the way that parents and children discuss health and illness. Consequently, it may be useful to apply LCA to explore possible patterns in children’s questions and parental responses about health and illness. While past research has described what topics children ask about and how parents respond (e.g., Menendez et al.; [3]), LCA could enable us to understand how the topics of conversations might cohere and whether the pattern of conversations across individuals can be categorized into a discrete number of classes.

### Current study

We examined whether children’s questions and parental responses regarding the COVID-19 pandemic obtained during the second peak (July 29th – August 10th, 2020) followed a similar pattern as those reported in previous studies conducted at an earlier stage of the pandemic [3]. More specifically, do children in the United States continue to ask questions about the changes in their daily routines during the second peak? Or are children seeking more information about the virus itself, its impact on the safety of themselves or family or friends, and/or the recommended preventive measures? Moreover, did parents in the current study continue to withhold information from their children by only providing yes/no/I don’t know type of responses to their questions with no further explanations?

Based on prior research, we anticipated that children would use questions to obtain information from their parents about the specific events related to the COVID-19 pandemic. The second peak of the COVID-19 pandemic happened approximately six months after the first verified case of COVID-19 in the United Staes [26–27]. The odds of families across the country contracting the virus themselves or someone they know had almost doubled [26–27]. Between the initial stage and the second peak of the pandemic, families had some time to adapt their routines and modify their lifestyles in response to the imposed restrictions. Consequently, we expected children to direct their questions to other pandemic-related events. In other words, we predicted that children’s questions about lifestyle changes will decrease as the pandemic progressed, but questions about the virus itself (e.g., what COVID-19 is and how it is spread) and preventative measures (e.g., stay-at-home orders and wearing masks) will become more frequent during the second peak than during the first peak.

Although we expect that there will be some changes in children’s questions and parental responses from peak one to peak two, it is not clear how other factors may influence the nature of children’s questions and parental responses over time. For that reason, we explored whether child factors (i.e., age, gender, biology knowledge, stress) and parental factors (i.e., biology knowledge, knowledge about COVID-19, level of education, stress) continue to influence the content of children’s questions and parental responses compared to previous studies conducted at the initial peak of the pandemic [3]. We did not anticipate that there would be any gender difference in the types of questions that children ask about the COVID-19 pandemic or the types of responses that parents provide. However, Halldorsdottir and colleagues [32] found that there were gender differences in the ways Icelandic boys and girls perceived the pandemic. Therefore, it is important to explore whether there are any gender differences in the questions children ask and/or the responses parents gave during the second peak of the pandemic in the United States.

A novel aspect of the current study is that, in addition to reporting on individual children’s questions and parental responses, we explored whether questions and responses could be classified into discrete groups (i.e., latent classes). This aspect of the study was exploratory in nature. To our knowledge, we are the first to use Latent Class Analysis (LCA) to classify children and parents into groups (i.e., latent classes) based on the overall pattern of questions and responses. It is important that we not only examine how parents might be responding to individual questions, but whether there are consistent patterns in both children’s questions and parents’ responses. This might provide us with a novel insight into the different styles of conversations that families in the United States engage in when discussing novel biological illnesses, such as COVID-19.

## Method

### Participants

This study was approved by the Research Subject Review Board of the University of Rochester (Institutional Review Board of University of Rochester, Study ID: 00005129, Date: 7.8.2020). Parents of 3- to 7-year-old children (N = 761) were recruited using Amazon’s Mechanical Turk. The sample size was based on previous work on this area [3,29] and with the expectation that some participants will be removed based on noncompliance. Participants also had to demonstrate a lifetime approval rating greater than 90%. Participants were presented with an electronic information sheet on Qualtrics that described all aspects of the study. An informed written consent was obtained from all participants before accessing the online survey. Participants completed the study on July 29^th^ through August 3^rd^, 2020, roughly a few months (March 1^st^ – May 31^st^, 2020) after several states and local jurisdictions declared stay-at-home orders and during the second peak of the COVID-19 pandemic [28,33]. Participants were paid $2 upon completing the survey. Two hundred and forty-five parents were omitted because of failure to pass at least one of our attention checks. The final sample resulted in 516 parents (M_age_ = 36.07, SD = 7.19, Age range 18–66). Participants came from 48 of the 50 states in the United States. Table 1 shows characteristic information for the final sample.

**Table 1:** Characteristic information of participants.

Characteristic	No. (%)
Parent Gender	
Male	195 (37.8)
Female	318 (61.6)
Unknown	3 (0.6)
Race/ethnicity	
Caucasian/White	402 (77.9)
Black or African American	46 (8.9)
Asian or Asian American	26 (5.0)
Hispanic	17 (3.3)
Native American	6 (1.2)
Latinx	1 (.2)
Middle Eastern	1 (.2)
Bi- or multi-race	10 (1.9)
Did not respond	7 (1.4)
Parent education level	
Some high school	1 (.2)
High school degree	38 (7.4)
Some college	68 (13.2)
Associate degree	65 (12.6)
Bachelor’s degree	233 (45.2)
Master’s degree	105 (20.4)
Doctoral level degree	6 (1.2)
Parent Age, mean (SD), years	36.07 (7.2)
Focus child age, mean (SD), years	5.14 (1.4)
Child gender	
Boy	294 (57.0)
Girl	222 (43.0)
MacArthur Perceived Social Status, mean (SD)	5.67 (1.9)
Parent COVID-19 knowledge, mean (SD)	5.40 (1.2)
Parent biology knowledge, mean (SD)	4.48 (1.2)
Child biology knowledge, mean (SD)	3.31 (1.5)
Anxiety, mean (SD)	
Parent	5.90 (2.9)
Child	3.74 (2.9)
Worry, mean (SD)	
Parent	6.16 (2.7)
Child	4.07 (2.9)

### Measures and procedures

**Survey.** The survey was based in part on a survey published in Menendez et al. [3]. Our survey included 155 questions that were administered through Qualtrics ® (Provo, UT). In this paper we focus on a subset of the total questions. We describe those in more detail below. The full survey can be found: https://rb.gy/mr8cxg.

To keep participants from knowing which responses were needed to access the full survey, these questions were randomized with additional distractor items. Participants who passed the screener were given a consent form to sign and were authorized to proceed to the survey.

**Attention Checks.** Two attention checks were presented within the survey. The first attention check was designed to ensure that the parents read the questions in their entirety. They were asked to indicate which news channel they typically watch, but at the end of the question they were asked to select “slightly disagree” regardless of which channel they actually watched. Participants who did not choose “slightly disagree” were not included in the study.

For the second attention check we asked parents to report on the age of the child they were focusing on at the beginning and end of the survey. This item was used to screen out participants who might not be truthful about their parental status and make sure that parents with multiple children were consistent on which child they were answering the survey about. We allowed a one-year difference between the age reported at the beginning and end of the survey to account for errors in typing. Participants who had a discrepancy larger than one year were excluded from the analyses. These sorts of attention checks are frequently used with online samples to filter out individuals who are completing the survey under false pretenses.

**Questions and Responses.** The current study focused on a subset of the total questions included in the broader survey. We primarily focused on the *types* of questions that parents reported their children asked them about the COVID-19 pandemic. To measure children’s questions, we asked parents to report up to *three questions* their children asked them about the events surrounding the COVID-19 pandemic. Parents were also asked to report how they responded to each of the three questions reported.

**Knowledge.** We asked parents to rate their own and their child’s understanding of biology using a 1 (Far below average) to 7 (Far above average) scale. Parents were also asked to answer if they “feel like [they] have enough knowledge or information to answer [their] child’s question about the COVID-19?” using a 1 (Strongly disagree) to 7 (Strongly agree) scale.

**Stress.** We asked parents to report “how *anxious* [they] (personally)/ [their child] are regarding the situation surrounding COVID-19” on a scale of 0 (not at all) to 10 (very). Parents were also asked to rate “how *worried* [they] (personally)/ [their child] are regarding the situation surrounding COVID-19” on a scale of 0 (not at all) to 10 (very). We combined these two questions to create a stress variable. We did so by taking the average of the ratings for both anxiety and worry.

### Qualitative coding

We coded the topic and content of children’s questions and parents’ responses by adapting the coding scheme reported by Menendez et al. [3]. Two independent coders coded all the responses. The reliability, measured through percent agreement and Cohen’s Kappa, was deemed appropriate for all categories (all Kappas were greater than.7). See Tables 2 and 3 for the coding schemes and reliability measures for each category. All disagreements were resolved through discussion. Throughout the paper, we report quotes from the written responses of our participants. These quotes have not been edited, and no grammar or spelling errors have been corrected in order to keep the data intact.

**Table 2:** Coding and frequency of the content of individual children’s questions.

Category	Description	Examples	Kappa	Percent Agreement	n (%)
Related to the virus	Questions about the nature of the coronavirus or the disease in general.	“What is COVID-19?” “How does coronavirus spread?” “When will it be gone?”	.89	94.8	350 (25%)
Safety of child, family, or friends	Questions about how the coronavirus may affect the child, family, or friend’s safety.	“Are my friends going to die?” “Am I at risk of getting sick?” “Is the world ending?”	.88	94.8	153 (11%)
Preventative measures	Questions about any preventative measures that have been recommended to slow the spread of COVID-19.	“Why do I have to wash my hands?” “Why do I have to use hand sanitizer?” “Why are people wearing masks?”	.98	99.3	186 (13%)
Lifestyle changes	Questions about lifestyle changes that came as a result of federal and state orders to slow the spread of COVID-19.	“Why can’t we leave the house?” “When can I see my friends?” “When can I go back to school?”	.95	98.1	588 (42%)
Other	Any question did not fit the categories listed above.	“No panic” “Eat healthy food”	.87	96.4	126 (9%)

**Table 3:** Coding and Frequency of the content of individual parent’s responses.

Category	Description	Examples	Kappa	Percent Agreement	n (%)
* No Explanation	Responses that do not provide an explanation	*“I’m not sure it might be fall”* *“Yes”* *“We don’t know yet.”*	.74	89.5	383 (27%)
*Authority	When the authority of parents, government officials or bodies, local authorities, or medical professionals, religious figures, and/or other authority figures that were not explicitly specified are used to answer the question.	*“that’s a decision their parents made up”* *“…wait until the doctors tell us it’s okay to”* *“As soon as the government says it’s safe.”* *“…and prayed up God will protect us”*	.71	89.1	330 (24%)
Address COVID-19	Responses that either specifically addressed COVID-19 or non-specifically addressed COVID-19 by using terms like “germs, illness, virus, sickness, and/or it”	*“Because they’re worried about the virus”* *“A virus like a cold”* *“No, but we wash our hands to kill any germs”*	.90	94.6	609 (44%)
Self-protection	Responses that encouraged the prioritization of the child’s and/or the family’s well-being/health	*“You can’t hug anyone besides us because they may have different germs”* *“To keep people around us safe”* *“It keeps us safe from the bad germs”*	.73	89.5	389 (28%)
Social Responsibility	Responses that promote the awareness of other people’s health and/or well-being	*“Schools are reopen when everyone getting recover from covid-19”* *“Do help stop the spread of the virus”*	.87	96.4	237 (17%)
Reassurance	If the parent tried to reassure the child.	*“…we will be okay…”* *“…don’t worry…”* *“…it will get better”* *“…we have nothing to worry about”*	.87	96.4	147 (11%)
*Supernatural	Responses that provide a supernatural explanation or personified the virus. Any reference to religious activities, practices, beliefs, or individuals that are typically seen as religious are included.	*“Yes, the Easter Bunny just hopped over the coronavirus…”* *“A tiny invader that attack’s a person’s body…”* *“The virus is very busy traveling around”*	.87	96.4	22 (2%)
Other	Responses that did not fit into any of the above categories.	*“Obama knew and tried to warn us about it”* *“avoid non-cooking food”*	.87	96.4	121 (9%)

(*) marks the codes that were mutually exclusive.

The “Supernatural” category was presented here separately, but due to its low frequency it was later consolidated into the “Other” category and was not part of further analysis.

Most of our coding categories were mutually exclusive for both questions and responses. However, some categories for parents’ responses allowed for multiple codes. For example, regardless of content, every response could also be classified as providing reassurance to the child.

### Analysis plan

Logistic regressions were conducted in R to explore whether the nature of children’s questions and parental responses differed across time points. Generalized mixed-effects models (with a binomial link) were conducted in R to examine whether the types of children’s question and parental responses were related to specific child and parental factors. The binary outcome was whether the child asked a specific question type (or not) and whether the parent provided a specific response (or not). All models examining individual children’s questions and parents’ responses included the child’s age, gender, biology knowledge, stress (the average of the ratings of child anxiety and child worry), as well as the parents’ biology knowledge, parents’ stress (average of the ratings of parental anxiety and parental worry), parents’ knowledge about COVID-19, and parents’ level of education as predictor variables. Since parents were allowed to report up to three questions and three responses, we included by-subject random intercepts. We then conducted Latent Class Analysis (LCA) using the poLCA package in R to examine whether children’s questions and parents’ responses could be characterized by a few discrete latent classes. We used Bayesian information criterion (BIC) and Akaike information criterion (AIC) to select the best models.

## Results

### Individual children’s questions and parental responses

On average, parents reported 2.7 child questions and 2.7 responses out of the three we asked them to report. Parents reported a total of 1,403 questions. Children’s questions generally sought information about lifestyle changes (42%), the virus itself (25%), and the preventive measures put in place to protect them (13%). Parents reported a total of 1,399 responses. Parental responses provided information about the virus (44%) and self-protection (28%). However, 27% of parents’ responses did not provide any explanations, often only providing yes/no/don’t know responses to children’s questions.

### Comparison of data collected at two different time points

To explore whether the pattern of children’s questions and parental responses changed over the course of the pandemic, we examined how the current results compare to those reported by Menendez et al. [3], who collected their data during the early part of the pandemic (April 14^th^-15^th^, 2020, first peak of infections and deaths in the United States). Our data was collected roughly around the second peak (July 29^th^-August 3^rd^, 2020). The samples did not differ on demographic variables examining parent and child biology knowledge, parent and child stress, or parental knowledge about COVID-19. It is important to note that Menendez et al. [3] recruited parents of 3-to-12-year-old children (M_age_ = 7.8, SD = 3.1) while for the current study we recruited parents of 3-to-7-year-old children (M_age_ = 5.14, SD = 1.4). More details are presented in S1 Table. The mean percentage of conversations initiated by children (40% vs. 44%) about the pandemic also did not significantly differ (*t* (794) = −1.84, **p* *= .07).

**Fig 1:**
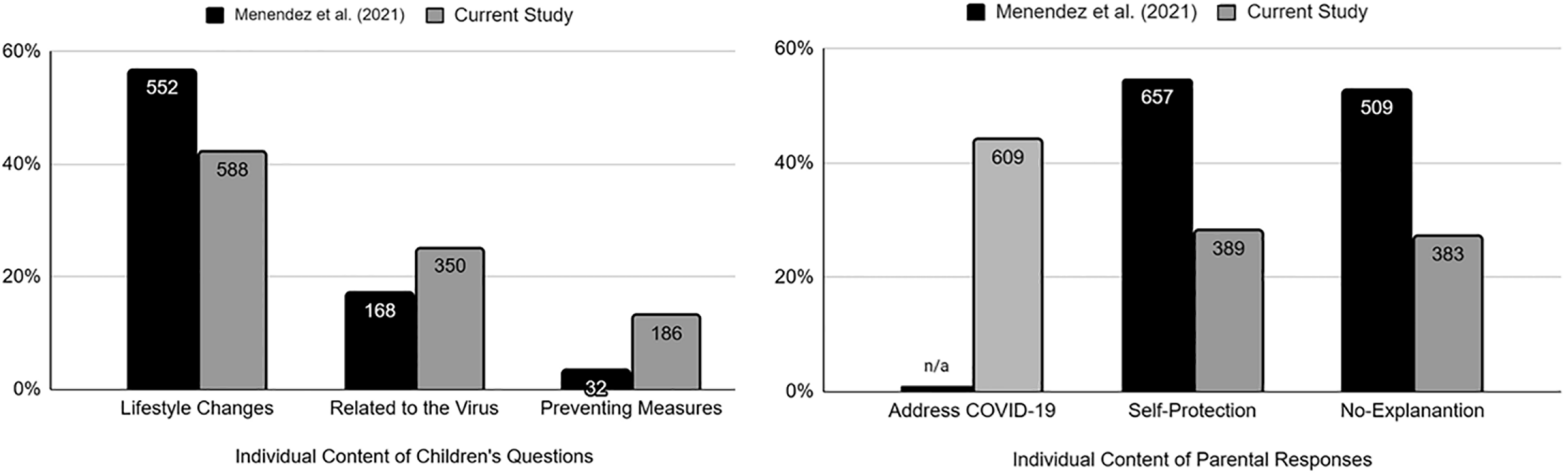
Comparing the most frequent questions and responses in Menendez et al. [3] and the Current Study. For the current study, we coded questions related to school or work under “Lifestyle Changes”, while for Menendez et al. [3] these were originally coded as separate. However, in this table we included questions related to school or work and interpersonal interaction as related to lifestyle changes for the Menendez et al. [3]. Also, in their study, Menendez et al. [3] did not code for parental responses addressing COVID-19 while for the current study we did as it was one of the most brought up responses. (Menendez et al. [3], N = 349; Current study, N = 516).

To explore whether the nature of children’s questions and parental responses differed across time points, we compared the most frequent types of questions and responses from this study to those described by Menendez et al. [3]. Fig 1 shows that lifestyle changes were the most common type of children’s question in both studies. However, results from a logistic regression analysis show that children in the current study were less likely to ask questions about lifestyle changes than those in Menendez et al. ( [3]; *OR* = 0.46, CI = 0.39–0.55, *p* < .01). On the other hand, results show that children in the current study were more likely to ask about the virus (*OR* = 1.6, 95%, CI = 1.3–1.9, *p* < .01) and the preventive measures put in place to protect them (*OR* = 4.5, 95%, CI = 3.1–6.7, *p* < .01) than children in Menendez et al. [3]. Overall, these results suggest that children in the second peak of the pandemic became more interested in various topics regarding the pandemic beyond just the changes in their daily routines.

By using logistic regressions, we were also able to find that parents in the current study were less likely to give their children yes/no/don’t know responses with no further explanations than parents in Menendez et al. [3] (*OR* =.34, 95%, CI = .28−.40, *p* < .01). Similarly, parents in the current study were less likely to report referencing self-protection in their responses to their children’s questions than in Menendez et al. [3] (*OR* =.32, 95%, CI = .27−.38, *p* < .01). Unlike in Menendez et al. [3], results show that the most common response that parents in the current study provided to their children were those that address COVID-19. This type of response was not coded in Menendez et al. [3]. Results suggest that, as the COVID-19 pandemic progressed, parents were more likely to provide responses that either specifically or non-specifically addressed COVID-19.

### Individual factors associated with individual questions

**Child Factors.** We used generalized mixed-effects models to determine whether children’s individual questions were associated with specific child and/or parental factors. We found that children who were rated by their parents as having lower levels of biology knowledge were more likely to ask questions about lifestyle changes (*OR* = 0.87, x^2^ (1, *N* = 1372) = −0.14, *p* < 0.01). Similarly, children who were rated by their parents as experiencing lower levels of stress were more likely to ask questions about lifestyle changes (*OR* = 0.88, x^2^ (1, *N* = 1372) = −0.12, *p* < 0.01). Despite not anticipating any gender differences, we found that boys were more likely to ask questions about preventive measures than girls (*OR* = 1.43, x^2^ (1, *N* = 1373) = 0.36, *p* = 0.03). We also found that younger children were more likely than older children to ask questions about preventive measures (*OR* = 0.89, x^2^ (1, *N* = 1373) = −0.12, *p* = 0.04). On the other hand, older children were more likely to ask questions regarding the safety of themselves, family, and/or friends (*OR* = 1.30, x^2^ (1, *N* = 1373) = 0.26, *p* = 0.01).

**Parental Factors**. We found that children with parents reporting higher levels of education were more likely to ask questions about lifestyle changes (*OR* = 1.19, x^2^ (1, *N* = 1372) = 0.18, *p* < 0.01). On the other hand, children with parents reporting lower levels of education were more likely to ask questions regarding the safety of themselves, family, and/or friends (*OR* = 0.78, x^2^ (1, *N* = 1373) = −0.24, **p* *= 0.04). Also, children with parents that reported lower levels of education were more likely to ask questions related to the virus (*OR* = 0.83, x^2^ (1, *N* = 1373) = −0.18, *p* = 0.01). See Table 4 for full results.

**Table 4:** Individual factors associated with individual children’s questions.

	Question Type (*OR*)			
Predictors	Related to the virus	Safety of child, family, or friends	Preventive measures	Lifestyle Changes
Child Age	1.09	1.30*	.89*	1.03
Child Gender	1.02	1.10	1.43*	.78
Child Biology Knowledge	1.13	.86	.90	.87***
Child Stress	1.04	1.09	.96	.88***
Parent Biology Knowledge	.96	1.15	.97	1.01
Parent Stress	1.02	.93	.95	1.04
Parent Knowledge about COVID-19	1.02	1.01	1.07	.93
Parent Level of Education	.83*	.78*	.97	1.19***

### Individual factors associated with individual responses

**Child Factors.** We also used generalized mixed-effects models to determine whether individual parental responses were associated with specific child and/or parental factors. Results show that parents who reported that their children were experiencing lower levels of stress were more likely to provide responses that addressed the COVID-19 virus or the pandemic (*OR* = 0.89, x^2^ (1, *N* = 1369) = −0.11, *p* < 0.01). Also, parents with children experiencing lower levels of stress were more likely to provide responses about self-protection (*OR* = 0.90, x^2^ (1, *N* = 1369) = −0.10, *p* < 0.01). Parents with children experiencing lower levels of stress were more likely to reference authority figures to answer their children’s questions (*OR* = 0.90, x^2^ (1, *N* = 1369) = −0.10, *p* < 0.01). Furthermore, parents who rated their children as experiencing lower levels of stress were also more likely to provide responses that promoted the awareness of other people’s health and/or wellbeing (*OR* = 0.85, x^2^ (1, *N* = 1369) = −0.17, *p* < 0.01). Parents who rated their children as experiencing lower levels of stress were more likely to provide reassuring messages to their children’s questions (*OR* = 0.89, x^2^ (1, *N* = 1368) = −0.12, *p* = 0.03). On the other hand, parents who reported their child as experiencing high levels of stress were more likely to provide responses with no explanations (*OR* = 1.10, x^2^ (1, *N* = 1368) = 0.09, *p* = 0.03). We found no significant differences in parental reports of children’s stress levels by gender.

Parents of younger children were also more likely to encourage the prioritization of the child’s and/or family’s well-being or health (*OR* = 0.89, x^2^ (1, *N* = 1369) = −0.12, *p* = 0.047). In contrast, parents with older children were more likely to provide reassuring messages to their children’s questions (*OR* = 1.33, x^2^ (1, *N* = 1368) = 0.28, *p* < 0.01). Parents with older children were also more likely to provide responses (e.g., yes, no, don’t know) with no explanations (*OR* = 1.29, x^2^ (1, *N* = 1368) = 0.26, *p* < 0.01). Also, parents of boys were also found to be more likely to provide responses that promoted the awareness of other people’s health and/or well-being (*OR* = 1.65, x^2^ (1, *N* = 1369) = 0.50, *p* = 0.02). Interestingly, we did not find any effects related to children’s biology knowledge.

**Parental Factors.** Parents who they themselves were experiencing high levels of stress were more likely to encourage the prioritization of the child’s and/or family’s well-being or health (*OR* = 1.10, x^2^ (1, *N* = 1369) = 0.09, *p* < 0.01). Parents reporting having completed higher levels of education were more likely to encourage the prioritization of the child’s and/or family’s well-being or health (*OR* = 1.16, x^2^ (1, *N* = 1369) = 0.15, *p* = 0.04). On the other hand, parents reporting less years of education were more likely to provide responses with no explanations

(*OR* = 0.82, x^2^ (1, *N* = 1368) = −0.20, *p* = 0.02). Those who did provide some sort of response were more likely to provide reassuring messages to their children’s questions (*OR* =.78, x^2^ (1, **N* *= 1368) = −.25, *p* = .02). Moreover, parents who rated themselves as having less COVID-19 knowledge or information were more likely to provide responses with no explanations (*OR* =.77, x^2^ (1, *N* = 1368) = −0.26, *p* < 0.01). On the other hand, parents who rated themselves as having more COVID-19 knowledge were more likely to reference authority figures (e.g., parents, doctors, government officials, religious figures) in their responses to their children’s questions (*OR* = 1.23, x^2^ (1, *N* = 1368) =.20, *p* = .02). We did not find any effects related to parents’ biology knowledge. See Table 5 for full results.

**Table 5:** Individual factors associated with individual parental responses.

	Response Type (*OR*)					
Predictors	No Explanation	Authority	Address COVID-19	Self-Protection	Social Responsibility	Reassurance
Child Age	1.29***	1.03	.92	.89*	1.03	1.33***
Child Gender	.8	1.31	1.28	1.22	1.65*	.7
Child Biology Knowledge	.94	1.02	.97	.92	1	.89
Child Stress	1.10*	.90***	.89***	.90***	.85***	.89*
Parent Biology Knowledge	1.21	.87	1.01	.99	.93	1.03
Parent Stress	.95	1.01	1.06	1.10***	1.08	1.07
Parent Knowledge about COVID-19	.77***	1.23*	1.06	1.04	1.14	1.01
Parent Level of Education	.82*	1.09	.99	1.16*	1.04	.78*

### Latent Class Analysis (LCA) of children’s questions and parental responses

Overall, we obtained a total of 501 parents with complete questions and responses. Fifteen parents were dropped from the analysis due to missing information about their children’s questions and/or parental responses. Due to the fact that the content of children’s questions and the content of parental responses involved different categories, we could not combine them in a single analysis Thus, we fitted different LCA models for children’s questions and parents’ responses, yielding one to five latent classes for children’s questions and one to six latent classes for parental responses. It is suggested that the BIC is the most reliable indicator for class selection, with lower values indicating better fit [34]. Using BIC as our criteria, the model with three classes had the best fit for both children’s questions (BIC = 2486.6) and parental responses (BIC = 3777.7). These three classes also made conceptual sense for both children and parents. Information on model comparisons is presented in S2 Table.

For children’s questions, we defined the three latent classes as: (1) primarily focusing on the virus [**n* *= 196, 39%], (2) focusing on both the virus and lifestyle changes [**n* *= 105, 21%], and (3) focusing on both lifestyle changes and preventive measures [**n* *= 200, 40%]. See Table 6 for examples of these different classes for children’s questions. For parents’ responses we defined the three latent classes as: (1) focusing on providing information about the virus itself and self-protection [*n =* 253, 54%], (2) focusing on reassurance while providing some information about the virus [**n* *= 162, 28%], and (3) providing no-explanation [e.g., yes/no/don’t know; **n* *= 86, 17%]. See Table 7 for examples of these different classes for parental responses.

**Table 6:** Latent Classes of Children’s Questions.

Latent Classes for Children’s Questions			
Latent Classes	The Virus	The Virus and Lifestyle Changes	Lifestyle Changes and Preventive Measures
*Examples*	*“When will the virus go away.”* *“why are so many people sick”* *“when the bad cold will be over”* *“When will the germs be gone?”*	*“Will a lot more people die?* *“What happens when you get corona?”* *“when is the virus going to be over?”* *“Why do i have to wear a mask?”*	*“Can i go to grand mother house”* *“When do I get to see my friends?”* *“Why everyone wearing mask?”* *“Why don’t I go to the store as much?”*

**Table 7:** Latent Classes of Parental Responses.

Latent Classes for Parents’ Responses			
Latent Classes	Information about the Virus and Self-Protection	Reassurance with some Information about the Virus	No-Explanation
*Examples*	*“we are home because of Cronavirus”* *“To keep people around us safe”*	*““You can catch it, but you’re young and healthy, so you probably won’t get it”* *“It is a very strong sickness and tons of people have it”*	*“Yes”* *“No”* *“good”*

### Factors influencing child and parent latent classes

Multinomial regressions were performed to determine whether parental and child factors predicted whether children and parents corresponded to a given latent class. In the models we included the child’s age, gender, biology knowledge, stress (the average of the ratings of child anxiety and child worry), as well as the parents’ biology knowledge, parents’ stress (average of the ratings of parent anxiety and parental worry), parents’ knowledge about COVID-19, and parents’ level of education to our models as predictor variables. Additionally, we compared all three latent classes for both children and parents separately by making each of the latent classes the reference group that was compared to the remaining two latent classes.

### Child latent classes

Children, who were reported by their parents to be, experiencing higher levels of stress were more likely to ask about both the virus and lifestyle changes than to ask about the virus (*OR* = 1.16, x^2^ (1, *N* = 491) =.14, *p* < .01) or about lifestyle changes and preventive measures (*OR* = 1.14, x^2^ (1, *N* = 491) =.13, *p* < .01), respectively. Moreover, children who were rated by their parents to have lower levels of biology knowledge were more likely to inquire about both lifestyle changes and preventive measures than about the virus (*OR* =.84, x^2^ (1, *N* = 491) = −.18, *p* = .04) or about both the virus and lifestyle changes (*OR* =.77, x^2^ (1, *N* = 491) = −.26, *p* < .01). Children with parents having obtained higher levels of education were more likely to ask about both lifestyle changes and preventive measures than to ask about both the virus and lifestyle changes (*OR* = 1.26, x^2^ (1, *N* = 491) =.23, *p* = .02). No other parental factors were found to differ between the child classes.

### Parent latent classes

Parents who reported having children with elevated levels of stress were more likely to provide no-explanations than information about the virus and self-protection (*OR* = 1.34, $\begin{array}{c} x \end{array}$^2^ (1, *N* = 491) =.30, *p* < .01) or reassurance with some information about the virus (*OR* =1.27, $\begin{array}{c} x \end{array}$^2^ (1, *N* = 491) =.24, *p* < .01). Parents who they themselves were experiencing high levels of stress were more likely to provide information about the virus than to provide no-explanations (*OR* = 2.84, x^2^ (1, *N* = 491) = 1.04, *p* = .04). No other factors were found to differ between the parent classes.

### Relating parent and child classes

**Fig 2:**
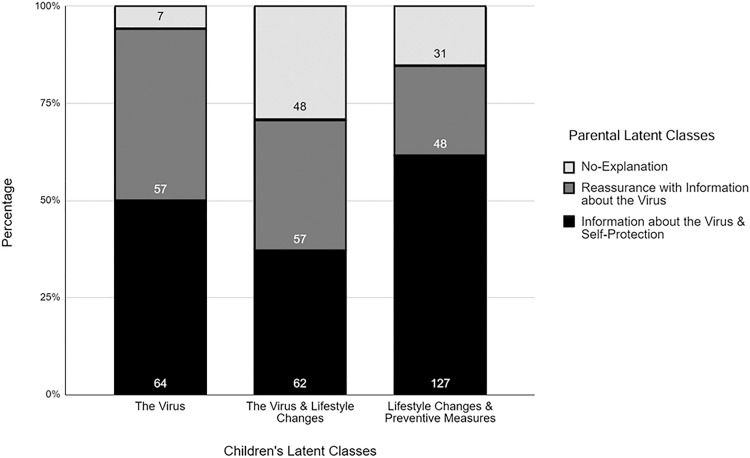
Number of children in each Child Class that had parents in each Parent Class.

We conducted a 3x3 cross-tabulation to explore how children’s latent classes relate to parental latent classes. As one can see in Fig 2, children who were primarily interested in learning about the virus were less likely to have parents who provided no explanations (*X*^2^ (1, *N* = 2) = 45.3, *p* < .01). Meanwhile, children who inquired about both the virus and lifestyle changes were equally likely to have parents from all three classes (*X*^2^ (1, *N* = 2) = 1.8, *p* = .4). Children who were interested in learning about lifestyle changes and preventive measures were more likely to have parents who provided information that was primarily about COVID-19 (*X*^2^ (1, *N* = 2) = 76.4, *p* < .01).

## Discussion

This study examined the nature of children’s questions and parental responses about the COVID-19 pandemic during the second peak of infections and deaths in the United States. An important contribution of this work is the examination of the stability of content of children’s questions and parents’ responses between the first and second peaks of the pandemic. The most important contribution of this work is the application of Latent Class Analysis (LCA) to examine patterns in children’s questions and parental responses. The majority of research on children’s questions and parents’ responses about the COVID-19 pandemic focuses either on individual questions and responses to those questions (e.g., [3–4]) or has focused on a small set of questions linked to causal reasoning (e.g., “what is coronavirus?” and “how someone gets coronavirus?”; [29]).

### Individual content of children’s questions and parental responses

Most of the prior work on children’s questions and parents’ responses about the pandemic were collected during the first peak in the United States. In the current study, we examined children’s questions and parental responses taking place during the second peak of the pandemic, a time of increased rates of infections and deaths due to COVID-19. We compared our results to those obtained during the first peak by Menendez et al. [3]. We found that child-initiated conversations about the events surrounding the pandemic occurred at similar rates in both studies.

We did find that the topic of children’s questions shifted between the two studies. Children’s questions about lifestyle changes became less frequent, questions about the virus itself (e.g., what COVID-19 is and how it is spread) and the preventive measures put in place to protect them (e.g., stay-at-home orders and wearing masks) became more frequent during the second peak than at the first peak of the pandemic. These changes likely occurred because children had become acclimated to the social changes and may have shifted their focus to learning more about the biological underpinnings of the virus. This difference in the topic of children’s questions may also be due to the different age groups in the two studies. As pointed out by Tõugu and colleagues [35], the sample in Menendez et al. [3] comprise of parents of slightly older children. Consequently, these older children may have experienced a wider disruption in their daily routines (e.g., going to school and having playdates) which ultimately led to them asking for more information about the changes in their daily life. Overall, these findings support the idea that children do ask questions to fill gap in their knowledge [17–19].

Moreover, we found that parents during the second peak of the pandemic were less likely to provide their children with yes/no/don’t know responses with no further explanations than parents in the first peak of the pandemic. This may reflect that parents may have had more knowledge about the virus and the effectiveness of preventive measures. Parental responses that encouraged the prioritization of the family’s well-being and health were less frequently reported by parents in the second peak compared to the first peak of the pandemic. This is somewhat surprising given the increase in the infection and death rates due to COVID-19 from peak one to peak two of the pandemic. It may be that having the preventive measures in place for a long-time lead to families having a sense of safety and security.

Children’s questions about the virus increased from peak one to peak two of the pandemic. This may explain why most parents focused on providing their children with information about the virus. Menendez et al. [3] took place at the early onset of the pandemic when parents did not know very much about the virus and appeared to be more focused on protecting their families from the virus. Thus, one major shift in the content of children’s questions and parental responses is towards children requesting more information about the virus itself and parents attempting to provide that information.

### Children’s questions

**Child Factors**. Children’s questions about the COVID-19 pandemic were found to be influenced by a number of child factors. For example, we found that children with lower levels of biology knowledge were more likely to ask about the changes in their lifestyle. Perhaps this is because the first major change brought about by the pandemic involved changes in daily routines. Viruses are complex biological phenomena that are difficult to fully understand even by adults [36–37]. Consequently, children may not have fully comprehended the pandemic itself. As a result, they may have focused on acquiring information for the changes in their daily lives. Moreover, Labotka and Gelman [5] noted that the children in their study were generally aware about the procedures they needed to follow in order to prevent the transmission of the virus; however, these same children did not understand the biological processes related to protective actions. Since viruses are complex biological phenomena, children with lower biology knowledge might have lacked understanding of the biological underpinnings of the pandemic. Thus, they focused more on the changes that they were experiencing as a result of it.

Similarly, children whose parents reported that their child was experiencing lower levels of stress were more likely to ask questions about lifestyle changes. This may have been because these children were less worried about the pandemic and getting sick. Therefore, they may have been more interested in why they were not able to do the things that they did before like visiting their loved ones or going to the park.

Parents in our study had children who were 3 to 7 years of age. Younger children were found to be more likely to ask questions about the preventive measures put in place to protect them and their families than older children. This may be the case because many medical and government officials recommended that families in the United States take extra precautions against the virus, such as wearing masks, which is not a common practice for most American children [38]. On the other hand, older children were found to be more likely to ask about the safety of themselves, family, and/or friends. The increase of infection and death rates and the implementation of stricter restrictive measures by some states may have caused older children to express concern as to how the events surrounding the pandemic might impact the safety and well-being of their loved ones. Future research should explore if or how the restrictive measures implemented may have influenced the types of questions children asked regarding the COVID-19 pandemic.

We found that boys were more likely than girls to ask their parents questions about the preventive measures put in place to protect them and their families. To our knowledge, there are no previous studies that show a gender difference in the types of questions children ask their parents in the domain of biological illnesses. However, some research examining gender differences related to children’s well-being during the COVID-19 pandemic [32] reported finding differences in the manner boys and girls perceived the pandemic. For example, when Icelandic adolescents were asked in what ways the COVID-19 pandemic affected their well-being, half of the girls reported that changes negatively impacted their daily routines compared to only 24% of the boys [32]. Therefore, the gender difference in the types of questions children in the United States asked their parents in the current study may be more of an indicator of the different ways girls and boys felt they were affected by the pandemic. It may also be due to the differences in the importance that girls and boys place on social activities and friendships [39].

**Parental factors.** We found a number of parental factors influencing children’s questions. For example, parents’ level of education was related to the types of questions children asked about the pandemic. Parents who reported having completed less years of education were more likely to have children that asked about the virus itself and the safety of themselves and their loved ones. Conversations between parents and children are not only driven by parents, but also by children and their questions [15–19]. In fact, we found that the average percentage of child-initiated conversations about the pandemic in the current study was of 44%. Children may be asking some of these questions because parents themselves fail to initiate and provide information about the COVID-19 pandemic simultaneously. In this manner, children may initiate important discussions with their parents in an attempt to fill gaps in their knowledge [17–19] about the pandemic.

### Parental responses

**Child factors.** Parental responses about the COVID-19 pandemic were also found to be related to child factors. During the second peak of the pandemic, parents who reported that their children experienced lower levels of stress were more willing to discuss various topics with their children surrounding the COVID-19 pandemic. This included information about the virus itself and explanations promoting the awareness of the health and well-being of the family. These parents may have felt more comfortable talking about the virus and health issues with their children. In contrast, parents who identified their children as experiencing high levels of stress were more likely to provide yes/no/don’t know responses with no explanations. Young et al. [22] state that parents can act as gatekeepers of information, managing, what, when, and how they share information with their children. The parents in the current study perhaps did not want to further distress their child, so they may have avoided giving explicit details to their children’s questions.

Parents of older children were found to be more likely to provide yes/no/don’t know responses and no further explanations to their children’s questions. Parents who did provide their older children with some sort of explanation often provided reassuring messages. Older children often asked about the safety of themselves and loved ones; thus, parents may have wanted to provide reassurance to them in hopes of avoiding worrying their child. On other hand, parents of younger children were more likely to give responses that encouraged the prioritization of the family’s well-being and health.

Previous studies have found that it can be very difficult for adults to explain scientific ideas to young children in a way that they can understand [35,40]. Consequently, some parents may choose to limit the amount of information they share with the child based upon whether they think their child is old enough to know about the topic [24]. Prior research on chronic illnesses in particular identifies the age of a child as one of the main determinants of what type of information is conveyed by parents [23–25]. As anticipated, when faced with a complex and unknown disease like COVID-19 was at the time data was collected, parents may resort to using the child’s age to determine what or how much information to share with the child. It may have been that parents of younger children perceived that their child may not have had the sufficient training and socialization about the most effective methods for reducing the spread of viral illnesses (e.g., proper hygiene like handwashing) like children of school-age do. Therefore, parents of younger children may have felt that they needed to encourage them to partake in behaviors that will protect the well-being and health of the family. It may have also been the case that parents of younger children were more willing to answer their questions because their questions tended to focus on preventive measures.

Moreover, parents of boys were found to be more likely to give responses that promoted the awareness of other people’s health and/or well-being. This may suggest that parents in the current study thought boys needed greater emphasis on social responsibility. Another plausible reason, as pointed out previously, gender differences in the types of responses may have been due to the different ways that boys and girls felt they were impacted by the pandemic [32]. Future research should take a closer look at factors that are associated with the different types of responses parents provide to boys versus girls.

**Parental factors.** Parental factors, such as parental education and stress, were found to influence the specific responses that parents gave to their children. For example, parents reporting that they themselves were experiencing high levels of stress were more likely to give responses that encouraged the prioritization of the health and well-being of the family. These parents may have been worried about the family and felt a need to encourage their children to follow specific practices and behaviors that would ensure the well-being of the family.

Similarly, more highly educated parents were more likely to provide explanations that emphasized the importance of prioritizing the health and well-being of the family. These parents may have perceived that they had the adequate knowledge to answer their children’s questions. On the other hand, parents who reported having completed less years of education were more likely to provide yes/no/don't know responses with no further explanations. Those who did provide some sort of explanation often provided reassuring messages. Prior research has shown that parents do not always provide thorough responses [20–24]. It may be that parents with less years of education may feel that they are not equipped with the appropriate knowledge or information to answer their children’s questions [20]. Consequently, they may choose to comfort and reassure their children that everything would be okay to avoid worrying them.

Moreover, parents who rated themselves as not having the adequate COVID-19 knowledge were more likely to provide no explanations to their children’s questions. On the other hand, parents who rated themselves as having the adequate COVID-19 knowledge provided children with some sort of explanation referencing authority figures (e.g., themselves, doctors, government officials, and/or religious figures) as part of their explanations to children’s questions. Thus, parents who are more knowledgeable tend to provide support for the validity of information given by authority figures.

### Latent classes of children’s questions and parental responses

One of the goals of the current study was to examine if Latent Class Analysis (LCA) could reveal distinct patterns of children’s questions and parental responses. The use of LCA provides a more holistic description of questions and responses, and a greater context for how individual questions fit into a larger conversational milieu. Our work shows that both children’s questions and parental responses can be categorized into discrete patterns. Furthermore, the patterns of parental response can be meaningfully related to the patterns of children’s questions.

Children’s questions were categorized into three latent classes that were about: (1) the virus, (2) the virus/lifestyle changes, and (3) lifestyle changes/preventive measures. Most of the children in the current study were split between groups one and three. Similarly, parental responses were divided into three latent classes: (1) information about the virus/self-protection, (2) reassurance/the virus, and (3) no-explanation. About half of the parents in the study belonged to group one.

This approach goes well beyond reporting the frequency of certain types of children’s question and parental responses. Although this work was exploratory, this approach may be useful in encouraging future research to go beyond looking at the individual content of questions and responses. By using LCA we are not merely reducing the number of categories of information, but we are demonstrating how we can group children who asked similar types of questions together and parents who answered questions in a similar way. Furthermore, we can see whether children’s latent classes and parental latent classes map on to each other in meaningful ways.

### Factors associated with child and parent latent classes

#### Child latent classes.

As with individual children’s questions and parental responses we found that some child and parental factors were associated with the child latent classes that children corresponded to. For example, children who experienced higher levels of stress were more likely to ask questions about the virus/lifestyle changes. On the other hand, children who were rated by their parents as having lower levels of biology knowledge were more likely to ask about lifestyle changes/preventive measures. Myant and Williams [8] found that children could not accurately explain the biological basis of contagion until they age 9 to 10. Additionally, Toyama [9] indicates that children’s conceptualization of illness diverges and becomes increasingly sophisticated in older childhood. Parents in our study had children who were 3 to 7 years of age and, according to these prior findings, have not yet reached a fully sophisticated understanding about illness and its causes. Consequently, due to their lower understanding of biology, children in this group may have focused their questions on aspects of the pandemic that are observable (e.g., people wearing masks) than on its biological underpinnings that are not observable with the naked eye.

#### Parent latent classes.

Furthermore, we found that both child and parent factors were related to the parent latent classes parents corresponded to. For example, parents of children experiencing elevated levels of stress were more likely to provide yes/no/don’t know answers with no further explanations. These parents may have chosen to limit the amount of information they share with their child due to perceived stress. This might be a form of shielding their children from information parents may think might upset their children. These findings are consistent with what was found for the individual parental responses. This group of parents were acting as gatekeepers of information, managing how much information they share with their children [22].

Furthermore, parents who reported higher levels of stress were more likely to provide information about the virus/self-protection. According to McIntosh and colleagues [13], children learn about the causes of illnesses through their participation in everyday family activities. Parents, through everyday conversations and explanations (or lack thereof) they give to their children, shape their child’s knowledge of biological illnesses. These conversations, in turn, may help shape the preventive behaviors children decide to engage in. Therefore, parents experiencing elevated levels of stress may be trying to reduce their own stress levels by having conversations with their children where they encourage them to engage in specific preventative behaviors to promote family health and well-being.

### Relating the patterns of child questions and parental responses

We also examined how the patterns of child questions related to the patterns of parental responses. We found that more than half of the children that sought information about lifestyle changes/preventive measures had parents that were in the latent class that tended to provide information about the virus/self-protection than any of the other two groups of parent latent classes. Similarly, children who asked only about the virus and the virus/lifestyle changes were more likely to have parents who provided information about the virus/self-protection than parents that provided reassurance/the virus or provided yes/no/don't know responses with no further explanations.

In the larger context of this study, one of the implications of these results is that there is not a perfect match between what children ask and how parents respond. It appears that parents are attempting to scaffold their children to mostly think about information about the virus and ways to protect them from it. Children on the other hand prioritized lifestyle changes and obtaining information about the virus.

### Limitations

Our study is limited by the fact that it is based on parent self-reports. While parental reports are subjective, they are perhaps the best source of information for infrequent child behaviors. While we do know from our study and previous research [3–5,29] that children do ask questions about COVID-19, but these questions are relatively infrequent. At the same time these questions are likely highly salient to parents. This salience might make these questions highly memorable. Additionally, even if parents do not remember the exact questions that their children asked and their exact responses, their reports likely reflect their perceptions of key issues that arose in their conversations. Although one could use a prospective dairy approach to examine parents’ reports of children’s questions, this approach is very time consuming and may not be practical for relatively infrequent behaviors, or events such as the pandemic that had uncertain timelines. Likewise, other methods for eliciting children’s questions using parent-child book reading or laboratory settings may not capture children’s spontaneous questions or be practical during a pandemic. Moreover, spontaneous children’s questions may reflect more what children want to learn than what may be elicited in a book reading setting.

It may be the case that the salience of certain questions may vary as a function of parental characteristics. For example, highly educated parents might find questions about the virus more salient and interesting than questions about behavioral changes. Future research might explore what factors influence the salience of children’s questions to parents. Understanding how parental characteristics might influence the salience of their children’s questions could be an interesting issue to explore in future researcher.

Moreover, the no explanation category and, later on, class of parents provides valuable insight into the style of parental responses. However, the results of this study could have benefited from further refining this category into potential subcategories. A distinction between answers that are driven by uncertainty (“I don’t know”) as opposed to attempts to reassure (“Hopefully soon”), for instance, could reveal different underlying motivations. However, the majority of responses in this category were yes, no, I don’t know responses (62%). Only 4% of the responses in this category could be potentially categorized as reassuring messages, such as “Hopefully soon.” The rest of the responses in this category were things like “Germs” and “Overseas”. Future studies may investigate the nuances that lie in parental responses such as the no explanation responses in order to increase our understanding of what strategies parents may be using to address their children’s complex questions about health and illness.

Another limitation of our study is that our sample was predominately White, highly educated, and mostly from a middle-class background. Although we have some representation of parents from other racial and ethnic groups, our sample was not fully representative of the United States population. Our sample was not likely as impacted by the pandemic as other more diverse groups in the United States who experienced higher rates of infections and deaths [26]. Clearly, the conversation in those families may be different from the ones that we captured.

## Conclusion

Our findings provide insight into the patterns of children’s questions and parental responses about health and illness. Children ask questions to fill gaps in their knowledge [17–19] which helps them build more detailed mental models about illnesses. Nevertheless, children’s conceptualization of illnesses cannot be understood without examining parental responses. Children’s perceptions of illness are influenced by the information that parents provide, which can encourage or discourage certain behaviors (e.g., proper hygiene).

Most previous research used a snapshot in time to capture conversations between parents and children. Our study captured the shift in dynamics in children’s questions and parental responses about the pandemic, showing a change in these conversation from the first to the second peak of infection and death rates in the United States. As COVID-19 infections and deaths increased, the number of questions children asked about the changes to their daily routines decreased and their questions about the virus and preventive measures increased. Over this period of time parents provided more detailed explanations about the virus likely as a consequence of society learning more about the virus.

A novel aspect of this study is the demonstration that LCA can be an effective tool to examine children’s questions and parental responses in a more holistic manner. We uncovered three distinct patterns related to children’s questions and parents’ responses and explored how they were related to one another. This enabled us to explore in more detail than past research the overall context of children’s questions and parents’ responses about COVID-19.

Our results have implications for health care professionals. These individuals need to understand what children know and do not know in order to help parents and children understand why they should engage in certain behaviors and what situations to avoid in a health crisis. Health care professionals also need to inform parents about the virus and protective measures so they can better provide information to their children. Consequently, when we encounter another global health crisis in the future, we will have some insight into how families in the United States might discuss novel illnesses. This would give us a better opportunity to prepare children to make proper health-related decisions. Finally, our results highlight the importance of obtaining multiple windows into children’s questions and parental responses to understand the processes of cognitive development.

## Supporting information

S1 Table Comparison of key demographic characteristics between Menendez et al. (2021) and the Current Study. (DOCX)

S2 Table Best model fit for children’s questions and parents’ responses. (PDF)
