# Supplementary material for: “What happens when you get corona?”: Children’s questions and parental responses about the COVID-19 pandemic
Source: PLoS One. 2025 Aug 18;20(8):e0330506. doi: 10.1371/journal.pone.0330506 (PMC12360530; doi:10.1371/journal.pone.0330506)
Supplement: S1 Table — (DOCX) [file pone.0330506.s001.docx]

|  | **Characteristics** | | | | | | |
| --- | --- | --- | --- | --- | --- | --- | --- |
|  | **Parent Biology Knowledge**  **Mean (SD)** | **Child Biology Knowledge**  **Mean (SD)** | **Parent Stress**  **Mean (SD)** | **Child Stress**  **Mean (SD)** | **Parent COVID-19 Knowledge**  **Mean (SD)** | **Child Age Years**  **Mean (SD)**  **[Range]** | **Mean (SD) percent of conversations started by the child** |
| **Menendez et al. (2021)**  **N = 349**  **Conducted:**  **April 14^th^ – 15^th^, 2020** | 4.5  (1.2) | 3.4  (1.4) | 6.3  (2.6) | 3.8  (2.7) | 5.6  (1.1) | 7.8  (3.1)  [3-12] | 39.7  (30.1) |
| **Current Study**  **N = 516**  **Conducted:**  **July 29^th^ – August 3^rd^, 2020** | 4.48  (1.2) | 3.31  (1.5) | 6.0  (2.7) | 3.9  (2.8) | 5.4  (1.2) | 5.14  (1.4)  [3-7] | 43.6  (33.2) |
