# Supplementary material for: “What happens when you get corona?”: Children’s questions and parental responses about the COVID-19 pandemic
Source: PLoS One. 2025 Aug 18;20(8):e0330506. doi: 10.1371/journal.pone.0330506 (PMC12360530; doi:10.1371/journal.pone.0330506)
Supplement: S2 Table — (PDF) [file pone.0330506.s002.pdf]

	**Model Fit**					
	Children's Questions			Parents' Responses		
Number of Classes	*AIC*	*BIC*	*Entropy*	*AIC*	*BIC*	*Entropy*
1	2514.42	2531.31	2.49	3887.05	3912.37	3.86
2	2448.90	2486.90	2.41	3736.07	3790.92	3.70
3	2427.50	2486.57	2.38	3693.37	3777.74	3.64
4	2427.42	2507.65	2.37	3683.36	3797.26	3.62
5	2433.40	2534.74	2.37	3678.24	3821.67	3.60
6	-	-	-	3685.29	3858.26	3.59
